# Omentum in the Pediatric Umbilical Hernia: Is It a Potential Alarm for the Appearance of Complications?

**DOI:** 10.1155/2012/463628

**Published:** 2012-11-07

**Authors:** Xenophon Sinopidis, Antonios Panagidis, Vasileios Alexopoulos, Ageliki Karatza, Konstantina Mitropoulou, Anastasia Varvarigou, George Georgiou

**Affiliations:** ^1^Department of Surgery, Karamandanion Children's Hospital, 26331 Patras, Greece; ^2^Department of Pediatrics, University General Hospital of Patras, 26504 Patras, Greece

## Abstract

Umbilical hernia is a common benign condition which resolves spontaneously during the first five years of life. However, in certain cases there are some characteristics which may be indicative of a different prognostic approach, as they increase the possibility of complications. The two cases of umbilical hernia that we describe here were treated operatively and revealed the presence of strangulated and adhered omentum, respectively. Reflecting on the adhesive properties of the omentum, we hypothesized that this may occur more often than it is believed, especially in those cases that are described as recurrent symptomatic herniations. In such cases, there should be increased alert for the possibility of complications during the period of the conservative expectance for resolution.

## 1. Introduction

Umbilical hernia is common in children and is diagnosed at birth. Spontaneous closure of the umbilical ring results in self-resolution of the hernia during the first years of life in the majority of cases [1]. Expectant management is recommended until the age of five years as a rule [1–3]. Complications are considered rare and are often documented in medical literature as brief reports or short series. Incarceration of omentum or abdominal hollow viscera into the umbilical ring is the most common mechanism implicated [4].

The operative findings of two umbilical hernia cases with strangulated and adhered omentum, respectively, posed a question on the role of the omentum as a prognostic factor: whether the presence of omentum into the umbilical hernia may be an indication of increased alert for the likelihood of complications and the performance of a prompt surgical repair during the period of the prolonged clinical observation.

## 2. Case Reports

**Figure 1:**
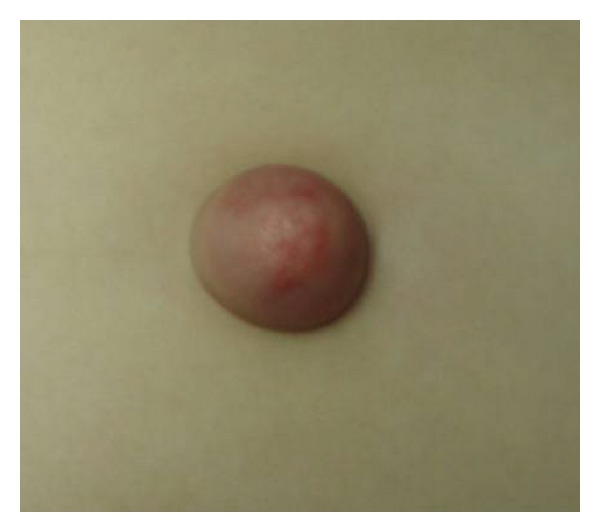
Clinical presentation of the incarcerated umbilical hernia (front view).

**Figure 2:**
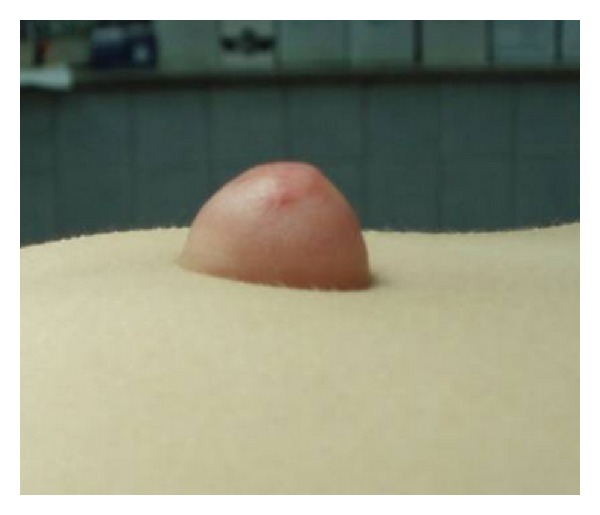
Clinical presentation of the incarcerated umbilical hernia (lateral view).

**Figure 3:**
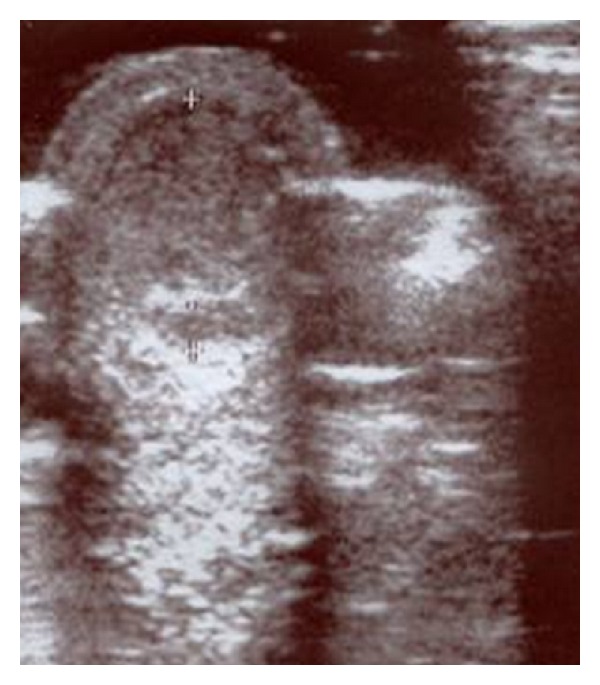
Ultrasound examination showing the presence of the omentum into the hernia.

**Figure 4:**
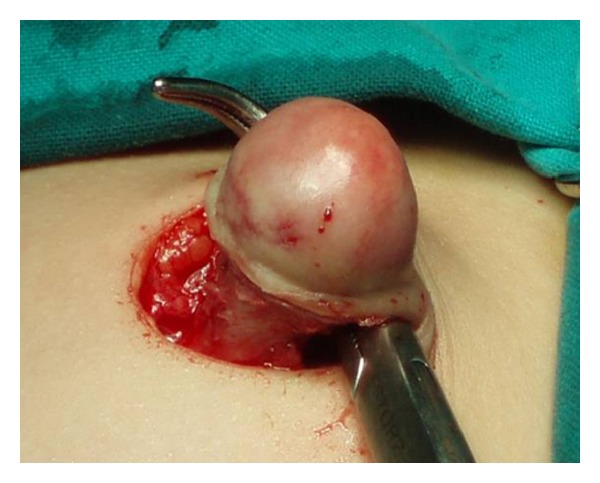
Operative picture showing the sac containing the strangulated and congested omentum.

 First case: a five-year-old female presented with an umbilical hernia. The hernia was present since birth. She was under periodical followup by her pediatrician, in anticipation of spontaneous healing. The hernia presented periodical size variation, often with palpable content. Abruptly, the hernia became tender and irreducible. The covering skin became red and edematous (Figures 1 and 2). Ultrasound examination showed incarcerated omentum through a 12 mm wide umbilical ring (Figure 3). Emergency surgery demonstrated the strangulated omentum (Figure 4). The congested omentum was adhered to the wall of the hernia. It was dissected free from the umbilicus and was reduced back to the peritoneal cavity. The umbilical opening was closed, and the patient had an uneventful recovery.

 Second case: a seven-year-old female presented with a history of an uncomplicated umbilical hernia. Persistence of the hernia to this age was an indication for surgical repair. There were no symptoms of strangulation, though the umbilicus was almost continuously bulky. During the operation, a tip of omentum was found firmly adhered to the hernia sac. It had to be dissected in order to be reduced into the peritoneal cavity. Closure of the umbilical ring followed, and the patient recovered well.

## 3. Discussion

Umbilical hernia is a common disorder of the anterior abdominal wall in children. Incidence is estimated approximately 15% with a 10–30% range in white children [2, 3]. Frequency is higher in children of African origin (85%), premature and small for gestational age neonates, patients with congenital hypothyroidism, Down syndrome, and patients undergoing peritoneal dialysis [2, 5–7].

 The umbilical ring closes before the age of four years in the majority of cases [1]. The tendency for spontaneous correction and the estimation that complications are rare created a benign profile for the umbilical hernia, leading the majority of western authors to recommend conservative therapy in children [3, 4]. The most common umbilical hernia complications are incarceration and strangulation which result in ischemia of the visceral contents. Foreign body impaction, gangrene, perforation, fistula formation, and evisceration have also been reported [2–4, 8]. 

 The overall incidence of complications in the western literature is regarded low. It is estimated by some researchers approximately 0.07% [6, 9]. In a study on 377 patients, the incidence of incarceration and strangulation was 8% [10]. Papagrigoriadis reported a rate of 1 : 1500 children [2]. A recent large study from the Mayo Clinic showed a relatively greater (7%) frequency of complications requiring emergent surgical repair [3]. Incidence is higher in studies of African origin [4, 6]. Strangulated hernias were found in 15% of all operations for umbilical hernia in a report from Senegal, where every child diagnosed to have an umbilical hernia is operated [11]. In a study from Nigeria on 47 patients operated for umbilical hernia, the complicated cases were 64% [4]. 

The greater omentum is regarded by some authors as the second most common content in complicated hernias after the small intestine [2, 9, 11]. Incarceration and strangulation of the omentum produce discomfort and pain. Cooper and Ferzoco regard the greater omentum as the most frequent content of any umbilical hernia, mention that it can pose a serious risk to the patient if strangulated, and should not be underestimated in considering urgent surgical repair [12]. Vrsansky supports that incarceration in children is much more frequent than supposed, and a more active therapeutic approach especially in smaller umbilical hernias is needed [13]. Recurrent abdominal pain in children with umbilical hernia has been attributed to recurring trapping of omentum within the hernia [14].  Table 1 demonstrates the variety of the umbilical hernia complications incidence and shows that our considerations are derived from a literature that is lacking a clear incidence of the omental content in an umbilical hernia (Table 1) [1–4, 9, 11, 13, 15–19]. 

With this knowledge in mind and after clinical observation, a question was raised: Is chronic or recurrent presence of omentum into the umbilical hernia a compromise to the conservative treatment and a factor increasing the possibility of incarceration? And if so, what is the mechanism? Our hypothesis is that the omentum may not enter and exit completely through the umbilical ring. Instead, a portion may remain adhered to the hernia wall, predisposing to recurrence or even to incarceration.

 The hypothesis is based on the omental physiology. Recent knowledge of the omental function provided information that differentiates it from the other hollow contents of a hernia [20]. The omentum is the primary peritoneal defense organ. It is responsible for the absorption and clearance of bacteria and debris from the peritoneal cavity. In response to foreign material or inflammation, it assumes an adhesive behavior to seal the impaired area. When the omentum is exposed to a stimulus, there is increase of the omental blood flow and expansion of the omental stromal tissue [20, 21]. Cells that express stem cell markers, inflammatory, and chemotactic substances such as the vascular endothelial growth factor and the fibroblast growth factor are produced [22–24]. The activated stromal cells engraft onto the irritated sites and lead to the recruitment of inflammatory cells within the peritoneal cavity. The result is the formation of adhesive bridges [20, 25, 26]. 

As the omentum enters into the narrow ring of an umbilical hernia it is subjected to mechanical pressure into a chamber of small volume. This mechanical stimulus may activate the inflammatory process and the formation of adhesions, resulting in the attachment of a portion of the protruding omentum into the hernia.

There are two interesting clinical options from this point on, which are represented by our two clinical cases. In the first case, strangulation is the most likely; if the abdominal pressure is instantly elevated, more omentum may enter into the hernia bulk, resulting in acute ischemia. The second option is the chronic or recurrent presence of omentum in the hernia as an adhered omental tip, functioning as a leading point. Interchange of empty and full hernia sac can be clinically inspected, guided by the permanent presence of the omental tip into the sac. In both incidences, indication for surgery is increased. Bain reported that spontaneous reduction after omentum incarceration happens in 86% of cases, assuming that incarceration may be more common than previously thought [27]. If an omental portion remains adhered into the umbilical hernia chamber, the increased frequency of recurrent incarcerations is explained by our hypothesis. When we discussed our theory of omental adhesions with experienced pediatric surgeons, they mentioned that in their operative practice, they have often noticed the presence of adhered omentum into the sac, which they induced back to the abdominal cavity before proceeding to the closure of the ring. 

We propose that clinical observation of the umbilical hernia in children should be more focused on the presence of the omentum. Ultrasound examination is helpful for diagnosis, especially in cases of umbilical hernias with recurrent pain. Timely surgical treatment should be under more serious consideration in these cases.

## Figures and Tables

**Table 1:** Reported cases and sac contents of incarcerated umbilical hernias in children.

Author	Reported complications of umbilical hernias	Contents of the umbilical hernia sac
Keshtgar and Griffiths [1]	7 cases with incarceration: 3 reduced without operation 2 of them with recurrent incarceration 2 of the cases were urgently operated	4 cases operated: 3 cases with small intestine 1 case with irreducible necrotic omentum

Papagrigoriadis et al. [2]	3 cases with incarceration	Small intestine (2) Small intestine and omentum (1)

Zendejas et al. [3]	34 cases, 7% of total umbilical hernias: Recurrent incarceration 22 Enteric fistula 7 Strangulation 4 Evisceration 1	Not specified

Ameh et al. [4, 15]	30 cases, 64% of total umbilical hernias: Acute incarceration 15 Recurrent incarceration 10 Evisceration 5	Specified only cases with gangrene: Intestinal gangrene (2) Omental gangrene (1)

Okada et al. [9]	1 reported case with strangulation Reviewed 38 cases with incarceration or strangulation	Terminal ileum, cecum, appendix Specified in the review: 34 Small intestine (20) Omentum (6) Terminal ileum and cecum (3) Transverse colon (3) Greater omentum and small intestine (2)

Fall et al. [11]	41 cases, 15% of total umbilical hernias Strangulation	Small intestine (27, 67%) Omentum (5, 12%) Small intestine, cecum, appendix (4, 9%) Nonidentified (5, 12%)

Vrsansky and Bourdelat [13, 16]	4 incarceration cases	3 with small intestine 1 with necrotic omentum

Chirdan et al. [17]	23 cases of incarceration, 44, 2% of total umbilical hernias: 15 with acute incarceration 6 with recurrent incarceration In 2 cases, parents declined surgery after reduction	1 case with gangrenous bowel with Meckel's diverticulum

Brown et al. [18]	28 cases with incarceration (7, 21% of all umbilical hernias repaired)	9 cases operated because of failed reduction of the content: 6 with omentum (2 necrotic) 3 with small bowel

Komlatsé et al. [19]	1 case of strangulation	Gangrenous Meckel's diverticulum
